# Fat talks first: how adipose tissue sets the pace of aging?

**DOI:** 10.1093/lifemedi/lnaf028

**Published:** 2025-07-19

**Authors:** Juanhong Liu, Qinlei Huang, Feng Liu

**Affiliations:** National Clinical Research Center for Metabolic Diseases, Metabolic Syndrome Research Center, The Second Xiangya Hospital of Central South University, Changsha 410000, China; National Clinical Research Center for Metabolic Diseases, Metabolic Syndrome Research Center, The Second Xiangya Hospital of Central South University, Changsha 410000, China; National Clinical Research Center for Metabolic Diseases, Metabolic Syndrome Research Center, The Second Xiangya Hospital of Central South University, Changsha 410000, China

**Keywords:** adipose tissue, aging, metabolic dysfunction, cell senescent, inflammation

## Abstract

Once viewed primarily as an energy reservoir, adipose tissue (AT) is now recognized as a key endocrinal organ in regulating systemic aging. With age, AT undergoes significant remodeling, marked by altered fat distribution, visceral fat expansion, impaired thermogenesis, and chronic low-grade inflammation, which disrupts metabolic and immune homeostasis. Emerging insights from single-cell and spatial transcriptomics highlight the critical roles of adipose progenitors, immune cells, and senescent cells in driving local dysfunction and systemic decline. Through inflammatory and metabolic signaling, dysfunctional AT actively contributes to age-related pathologies. This review explores how AT functions as both an early sensor and driver of aging and discusses therapeutic opportunities targeting adipose dysfunction to promote healthy aging.

## Introduction

With the dramatic increase in global life expectancy, populations worldwide are aging at an unprecedented rate. According to the World Health Organization, the global population aged 60 years and older reached approximately 1 billion in 2019 and is projected to grow to 2.1 billion by 2050, accounting for 20% of the total population [1]. Aging is a multifaceted biological process characterized by a progressive decline in physiological integrity, increased vulnerability to age-associated diseases, and eventual mortality [2, 3]. At the cellular and molecular levels, hallmarks of aging, such as genomic instability, telomere attrition, proteostasis decline, cellular senescence, and mitochondrial dysfunction, promote this process [2–6]. Despite significant advances in aging research facilitated by multi-omics technologies, the role of adipose tissue (AT) in orchestrating systemic aging remains an underexplored frontier.

AT holds a central role in aging due to its unique dual function. As the body’s primary energy reservoir and a dynamic endocrine organ, it not only regulates metabolism, hormonal balance, and immune responses but also exerts far-reaching effects on distant tissues through the secretion of diverse signaling factors. However, with advancing age, the plasticity of AT is progressively impaired, leading to profound biological changes such as increased visceral fat accumulation, reduced lipolysis and thermogenesis, and impaired ability to maintain core body temperature under cold stress [7–10]. These dysfunctions not only compromise the intrinsic roles of AT but also drive systemic metabolic and immune imbalances, inducing chronic low-grade inflammation, insulin resistance, and metabolic syndrome, all of which accelerate systemic aging [11–14].

Importantly, AT is hypothesized to be one of the earliest responders to aging signals. Studies have shown that age-related immune responses first emerge in white adipose depots of both mice and humans [5, 15], suggesting that AT may act as a key “trigger” for systemic aging by orchestrating metabolic-immune trade-offs. However, critical questions remain: Why is AT particularly vulnerable to aging? How do its secreted metabolic and immune factors orchestrate systemic aging through long-range signaling? Furthermore, the therapeutic potential of targeting AT to mitigate aging has yet to be fully realized. Recent technological advancements are beginning to shed light on these issues. For instance, single-cell and spatial transcriptomics have revealed the cellular heterogeneity of AT and the complex interactions between different cell types during aging [16]. Notably, adipose progenitor cells (APCs) begin to exhibit classical features of cellular senescence as early as midlife, characterized by the upregulation of p16, p21, IL-6, and IGFBP3. In parallel, extracellular vesicles (EVs) released from APCs display functional decline, contributing to immune dysregulation and the loss of AT homeostasis [17]. Notably, APCs play a dual role in immune and metabolic regulation, offering new insights into how AT acts as a “signal hub” to guide systemic aging. Emerging evidence also points to the role of adipose-derived exosomes and metabolic signals in influencing distant tissues, such as skeletal muscle and the central nervous system, potentially serving as key mechanisms for inter-organ communication during aging.

This review proposes that AT is not merely an early sensor of aging-induced changes but may function as a “central hub” driving systemic aging. By focusing on the characteristics of AT aging and its contribution to systemic aging, we aim to illuminate its unique position as a cornerstone of organismal aging and its potential as a therapeutic target. This perspective offers novel insights into the mechanisms of aging and paves the way for developing targeted intervention strategies to combat aging-related diseases.

## Changes in AT during aging

AT is a highly heterogeneous endocrine organ. The functional diversity of adipose depots arises from their distinct developmental origins and intrinsic metabolic, endocrine, and thermogenic properties [18]. In humans, AT is primarily classified into white AT (WAT) and brown AT (BAT). BAT is abundant in newborns, particularly in the interscapular region, while in adults, it persists mainly in the cervical region, supraclavicular fossa, and perirenal areas. Although BAT is much less prevalent than WAT in adults, it can be activated by cold exposure or specific hormonal stimuli. In mice, AT is functionally classified into WAT, BAT, and beige AT (bAT) [19]. WAT serves as the primary site for energy storage, sequestering excess energy as triglycerides in adipocytes during periods of surplus and releasing it during energy deficits to maintain systemic energy homeostasis [20, 21]. In contrast, BAT and bAT are thermogenic tissues capable of dissipating energy as heat, thus playing a crucial role in energy expenditure. Here, we focus on the functional and structural changes in these three AT types during the aging process.

### White adipose tissue

#### Redistribution and remodeling

**Figure 1.**
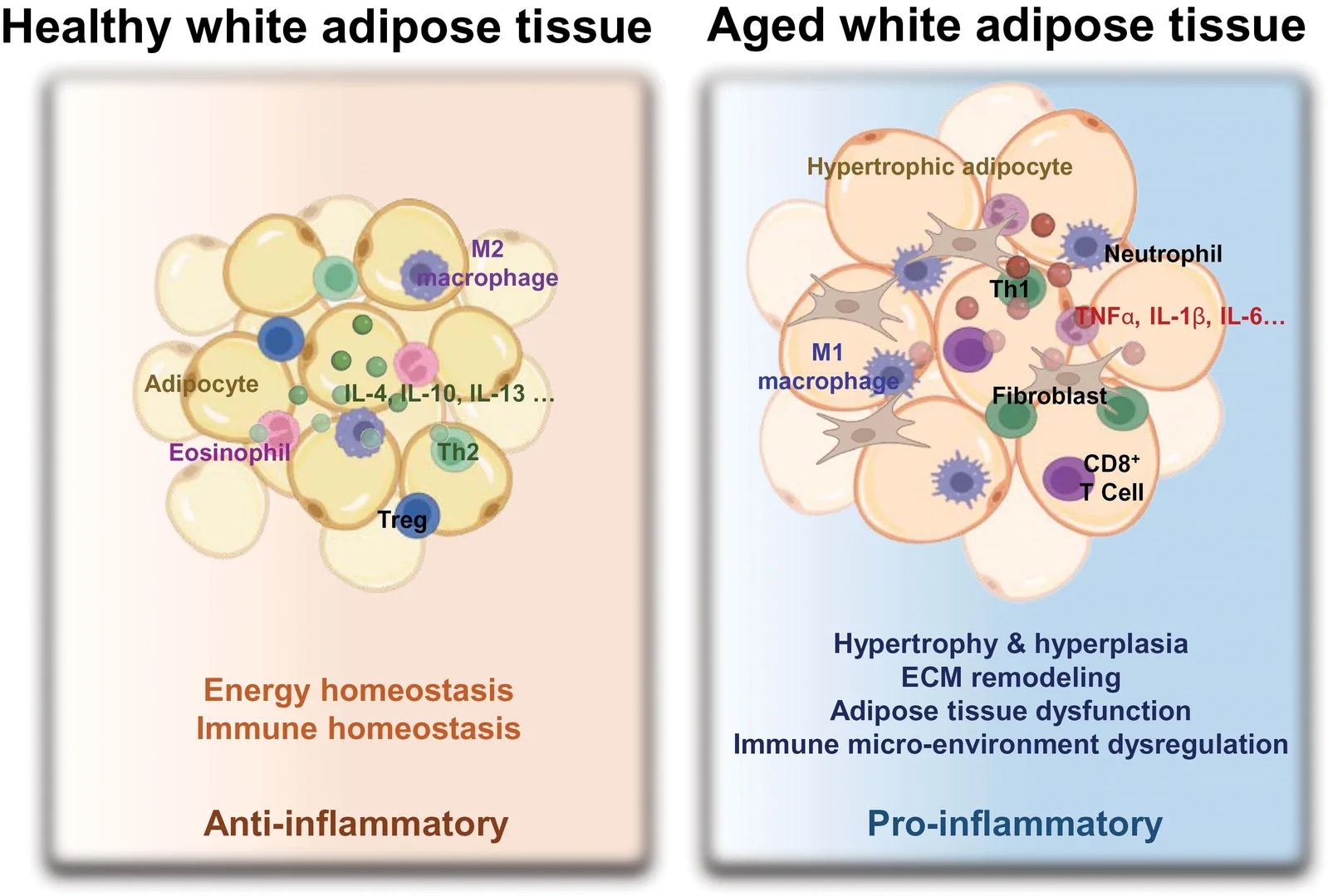
Characteristics of normal and aging white AT. Normal AT primarily consists of adipocytes and anti-inflammatory immune cells, including eosinophils, M2 macrophages, regulatory T cells (Tregs), and Th2 cells. These cells collectively maintain energy homeostasis and immune balance. In contrast, aging AT is characterized by hypertrophic and hyperplastic adipocytes, accompanied by stromal remodeling, fibrosis, and fibroblast infiltration. Pro-inflammatory immune cells, such as M1 macrophages, Th1 cells, and CD8^+^ T cells, dominate the immune microenvironment in aging AT. The accumulation of pro-inflammatory cells, combined with adipocyte dysfunction and immune microenvironment dysregulation, drives chronic low-grade inflammation and the establishment of a pro-inflammatory milieu. Additionally, stromal remodeling and fibrosis reduce the metabolic flexibility of AT, further exacerbating systemic metabolic dysfunction during aging. Created with BioRender.com, released under a Creative Commons Attribution-NonCommercial-NoDerivs 4.0 International license.

**Figure 2.**
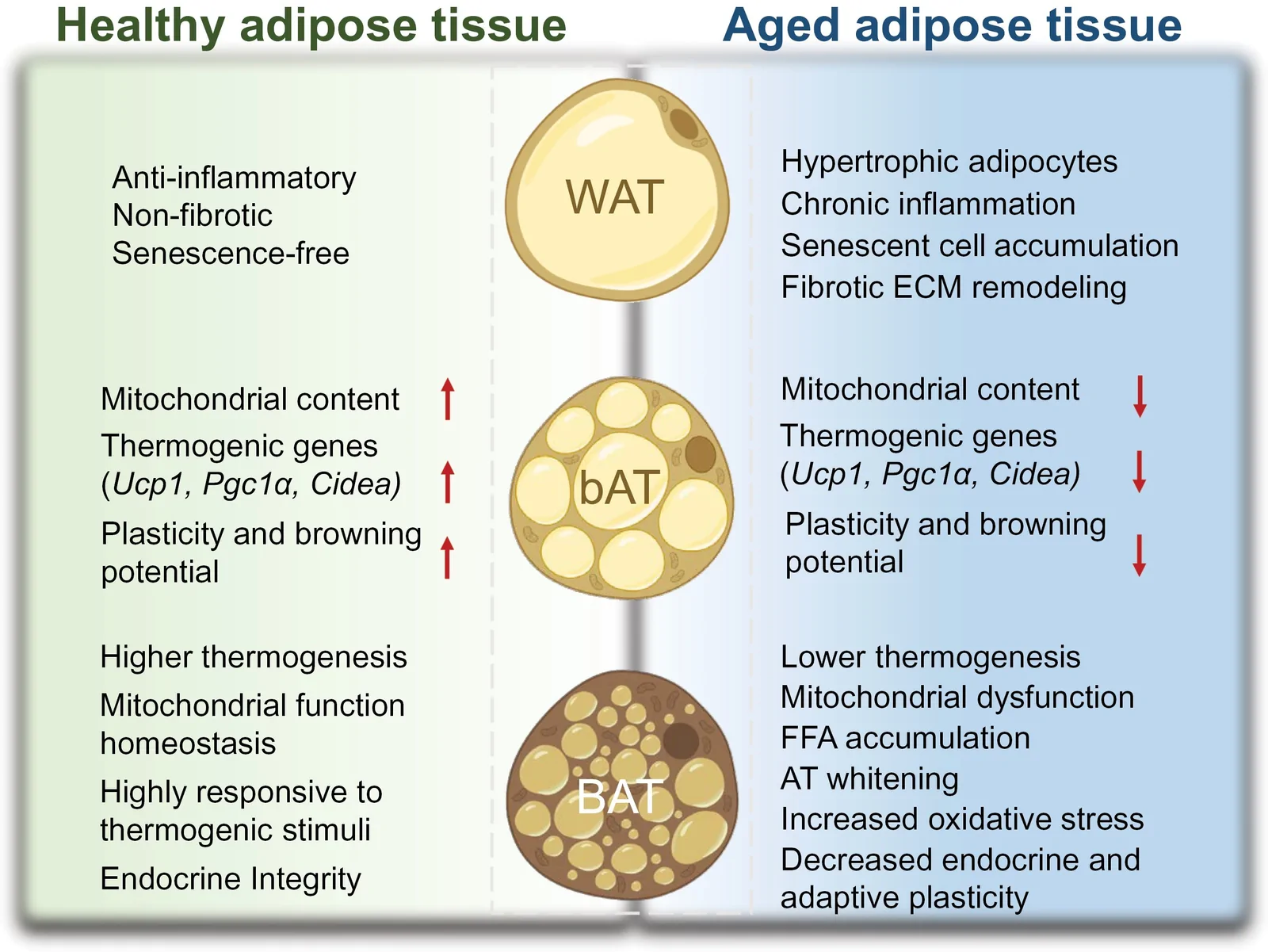
Comparative characteristics of healthy and aged white, brown, and beige AT. In healthy WAT maintains adipocyte size homeostasis, insulin sensitivity, and endocrine function, whereas aged WAT is characterized by adipocyte hypertrophy, increased pro-inflammatory signaling, extracellular matrix (ECM) fibrosis, and the accumulation of senescent cells, which together contribute to metabolic dysregulation and inflammaging. In healthy BAT and beige fat, adipocytes are rich in mitochondria and express high levels of UCP1, PGC1α, and Cidea, enabling robust nonshivering thermogenesis and energy expenditure. With aging, both BAT and beige fat show reduced expression of thermogenic genes, mitochondrial dysfunction, and decline in heat-producing capacity. Beige fat also undergoes structural “whitening”, marked by lipid droplet coalescence and loss of plasticity. Created with BioRender.com, released under a Creative Commons Attribution-NonCommercial-NoDerivs 4.0 International license.

WAT, the primary site of energy storage and endocrine regulation in the body, undergoes significant structural and functional remodeling during aging (Fig. 1). WAT is classified into subcutaneous AT (SAT) and visceral AT (VAT), which have distinct roles in metabolic regulation: SAT generally provides metabolic protection by improving insulin sensitivity and mitigating lipotoxicity, whereas VAT is closely associated with insulin resistance and chronic inflammation due to its secretion of pro-inflammatory factors such as TNF-α and IL-6. During aging, WAT distribution is markedly reshaped, characterized by an increase in VAT and a concurrent reduction in SAT [22]. In humans, aging is characterized by a progressive loss of SAT (particularly in the limbs and face), which leads to visible signs such as skin laxity and reduced mechanical cushioning [9, 23]. Concurrently, there is a marked increase in VAT, including fat surrounding the mesentery, kidneys, and abdominal organs, contributing to central obesity and elevated risk of metabolic syndrome, insulin resistance, and cardiovascular disease [24]. In rodent models, particularly mice, similar redistribution patterns have been observed. Subcutaneous fat depots such as inguinal WAT (iWAT) decrease in mass and show impaired adipogenic and browning potential. Conversely, visceral fat depots, including epididymal WAT (eWAT) and perirenal WAT, tend to expand with age and exhibit increased inflammatory infiltration, macrophage accumulation (predominantly pro-inflammatory M1 phenotype), and heightened expression of cytokines, paralleling the development of insulin resistance. This redistribution not only reflects the aging process of AT but also significantly elevates the risk of metabolic disorders such as type 2 diabetes and atherosclerosis, as well as cardiovascular diseases [25–29]. This remodeling is driven by multifaceted biological mechanisms, including the gradual decline in precursor cell functionality, reduced angiogenesis, and the exacerbation of pro-inflammatory environments (Figs. 1 and 2).

#### Hypertrophy and hyperplasia

Adipocytes expand as age-related weight gain progresses. In mice, total body fat reaches its peak during middle age, followed by a decline in later years, patterns that are also observed in humans [11]. Adipocyte expansion occurs in two ways: hypertrophic (an increase in adipocyte size) and hyperplastic (an increase in adipocyte number) [30]. Both are observed in the contexts of obesity and aging. Adipocyte size increases from middle age to older age, followed by a reduction in size in advanced aging [31]. An early study demonstrated that the canonical senescence markers Galactosidase Beta 1 (GLB1) and Tumor protein 53 (TP53) are significantly upregulated in aged human adipocytes [32]. Notably, the expression levels of these markers showed a strong positive correlation with adipocyte size, suggesting a potential mechanistic link between cellular hypertrophy and the activation of senescence pathways in AT during aging [32]. While hypertrophy enables AT to store more lipids, thereby protecting other organs from lipotoxic stress, it also reduces the surface area-to-volume ratio of adipocytes. This reduction impairs nutrient transport and cellular signaling, contributing to tissue dysfunction and metabolic imbalance. Furthermore, hypertrophic adipocytes secrete higher levels of pro-inflammatory factors, such as IL-6 and TNF-α, which induce chronic low-grade inflammation and exacerbate AT dysfunction.

#### Hypoxia and fibrosis

Hypertrophy may cause adipocytes to expand beyond the oxygen diffusion limit, reducing blood supply and promoting a hypoxic microenvironment. Hypoxia triggers hypoxia-inducible factor (HIF)-mediated responses, which upregulate angiogenesis to restore oxygen levels. However, this compensatory mechanism may be compromised in obesity, as hypoxic WAT exhibits reduced vascular density and lower levels of vascular endothelial growth factor A (VEGFA) [33]. Evidence suggests that AT hypoxia also occurs during aging [34]. Age-related hypertrophic expansion exacerbates oxygen diffusion limitations, while insufficient vascular compensation worsens the hypoxic state. Moreover, the angiogenic potential of adipose-derived stem cells (ADSCs) decreases with donor age, and both VEGF expression and vascular density in mouse AT exhibit age-dependent declines [35, 36]. These factors collectively intensify AT hypoxia.

While it remains unclear whether increased hypoxia is merely a byproduct of morphological changes associated with aging or whether hypoxic signaling actively drives AT dysfunction and the progression of metabolic disorders, the association between hypoxia and AT fibrosis is well established. Changes in vascular distribution and oxygen supply during AT expansion are accompanied by extracellular matrix (ECM) remodeling linked to fibrosis, and HIF-1α plays a central role in this process. Under chronic hypoxic conditions, HIF-1α induces the expression of profibrotic mediators such as lysyl oxidase (LOX), tissue inhibitor of metalloproteinases (TIMPs), and collagen isoforms (COL1A1, COL3A1), promoting ECM cross-linking and deposition [33, 37]. Collagen deposition, leading to fibrosis, has been observed in both obese mice and humans [33, 38]. Additionally, aging is associated with increased AT fibrosis, as evidenced by a sevenfold increase in collagen staining in the VAT of aged mice [36]. Hypoxia, a common feature of aging AT, promotes the expression of key inflammatory genes, including *IL-6* and *MIF-1*, through HIF-1α activation. Several studies have also reported a strong association between fibrosis and chronic inflammation [37, 39]. Collectively, these findings indicate that aging AT is characterized by a fibrotic and hypoxic microenvironment that contributes to local and systemic inflammation.

#### AT immune microenvironment

Under physiological conditions, the immune microenvironment of AT is primarily composed of regulatory anti-inflammatory immune cells, including alternatively activated macrophages, eosinophils, ILC2s, T regulatory (Treg) cells, invariant natural killer T (iNKT) cells, and γδT cells [40]. These cells secrete cytokines, such as IL-4, IL-10, and IL-13, establish an anti-inflammatory environment [41]. However, in obesity and/or aging, the immune microenvironment of AT undergoes a significant shift towards a pro-inflammatory state. This is characterized by an increase in M1 macrophages, CD8^+^ T cells, and other pro-inflammatory cells, while the number of Treg cells and eosinophils decreases [40]. This immune imbalance leads to the establishment of a chronic low-grade inflammatory environment, which is a key mechanism underlying the metabolic disorders induced by obesity.

In C57BL/6 mice, a short-term high-fat diet (HFD) specifically induces inflammation in AT, without triggering similar inflammatory responses in other metabolic tissues [42]. However, prolonged HFD feeding significantly enhances pro-inflammatory responses in other metabolic organs, including the liver and muscle [42]. This suggests that the inflammatory response caused by excess energy intake is initially triggered in AT, and chronic inflammation in AT subsequently spreads to other organs, ultimately leading to systemic metabolic dysfunction [30].

Obesity is a major risk factor for aging, which induces profound changes in the immune microenvironment of AT during aging. In the early stages of aging, the immune imbalance in AT, along with the associated changes in immune cell populations, is closely linked to the accumulation of pro-inflammatory macrophages and CD8^+^ T cells, along with a reduction in regulatory T cells [43]. This immune imbalance not only drives local chronic inflammation but also influences distant organs by releasing pro-inflammatory factors such as IL-6 and TNF-α, thereby promoting systemic inflammation and metabolic dysfunction.

A hallmark of aging in AT is the progressive increase in chronic, low-grade inflammation [44]. Accumulating evidence indicates that with advancing age, immune cell infiltration into adipose depots escalates, skewing toward a pro-inflammatory phenotype [45]. In older mice, expansions of cluster of differentiation 8 (CD8^+^) and CD4^+^ T cells have been documented in VAT [45]. In female mice, aging is further accompanied by an accumulation of resident B cells in AT [46]. Although the overall number of macrophages does not necessarily increase with age, there is a notable shift in macrophage polarization from an M2-like, anti-inflammatory state to an M1-like, pro-inflammatory state [45]. Additionally, senescent cells that accumulate in adipose depots contribute to this pro-inflammatory environment by means of the senescence-associated secretory phenotype (SASP) [47], in which they secrete a range of cytokines and chemokines that propagate local inflammatory signals systemically, thereby exacerbating “inflammaging” [48]. This chronic, widespread immune activation not only disrupts AT homeostasis but is also tightly linked to a variety of age-related pathologies, including cardiovascular and metabolic disorders.

### Brown and beige AT

The progressive depletion of all types of brown adipocytes during aging has been extensively documented in both rodent models and humans [49] (Fig. 2). BAT, which forms during gestation, plays a crucial role in thermoregulation, particularly in neonates who lack the ability to generate heat via shivering. Beige adipocytes, which differentiate from specific progenitor subpopulations in WAT, are believed to exhibit impaired proliferative and differentiation potential with age. The age-related decline in beige adipocyte numbers may be attributed to alterations in the AT microenvironment, particularly in the availability of nutrients and growth factors that modulate progenitor cell behavior. However, direct evidence to substantiate this hypothesis remains insufficient. Both the functionality and mass of BAT are known to decline progressively with advancing age, a phenomenon observed in various mammalian species, including rodents and humans [50, 51]. Accumulating evidence from computed tomography (CT) imaging studies indicates that the volume of BAT in humans declines progressively with advancing age [52]. In addition, BAT activity stabilizes around the age of 60, with a subsequent decline thereafter [53]. Notably, approximately 10% of individuals aged between 50 and 60 years retain metabolically active BAT [54]. Concurrently, aging leads to a reduced cold-induced activation of BAT, correlating with decreased cold tolerance and impaired thermoregulation in older adults [55]. In rats, the infiltration of white adipocytes into the interscapular BAT during aging markedly reduces uncoupling protein 1 (UCP1) activity, thereby compromising thermogenic capacity [51]. These findings reveal that aging exerts a profound impact on BAT activity, resulting in a nearly universal reduction as life progresses [56, 57].

## Secretory molecules and adipose aging

Adipokines are bioactive molecules secreted by AT that play important roles in regulating local and systemic energy metabolism, immune responses, and metabolic homeostasis [58]. Recent studies have revealed that adipokines not only contribute to energy metabolism and immune regulation but also significantly influence the aging process [59–61]. During aging, substantial changes occur in AT function and metabolism, which, in turn, alter the secretion patterns of adipokines (Table 1).

**Table 1.** Changes in adipokines in aged AT.

Adipokine	Change with aging	Aging-related role/mechanism
**Adiponectin**	↓Decreased [62]	Aging impairs adipocyte function and reduces adiponectin secretion, leading to increased insulin resistance and chronic low-grade inflammation.
**Leptin**	↓Increased [63]	Leptin levels rise with age due to adiposity, but leptin resistance develops, impairing central appetite regulation and promoting low-grade inflammation and metabolic inflexibility.
**PAI-1**	↑Increased [64]	Considered a hallmark of aging; elevated in aged AT, contributing to ECM remodeling, tissue fibrosis, inflammation, and senescence.
**RBP4**	↑Increased [65]	Overexpressed in aged or obese AT; linked to systemic insulin resistance, chronic inflammation, and age-related metabolic dysfunction.
**FGF21**	↑Increased [66]	Circulating FGF21 levels increase with age, but often accompanied by FGF21 resistance, reducing its efficacy in metabolic regulation despite elevated levels.

The trends presented in this table reflect general directions of change with aging (↑ increase, ↓ decrease, ↔ no consistent change), as reported across various studies. Due to substantial inter-individual variability and the influence of multiple confounding factors—including body composition, fat distribution, metabolic status, sex, genetic background, and lifestyle—it is currently not feasible to provide universally applicable quantitative values (e.g. fold changes) for each adipokine.

### Adiponectin

Adiponectin plays multifaceted and critical roles during the aging process. Accumulating evidence indicates that circulating levels of adiponectin decline with age, a reduction that is strongly associated with metabolic dysregulation, chronic inflammation, and cardiovascular pathologies in the elderly [67]. In addition, overexpression of adiponectin in aged mice significantly enhances glucose tolerance and insulin sensitivity, promotes lipid clearance, reduces visceral adiposity, and attenuates both inflammation and fibrosis, culminating in a metabolically healthy phenotype [67]. Given that tissue inflammation and fibrosis are widely recognized as critical determinants of lifespan, these findings position adiponectin as a promising candidate for combating aging and related disorders. Consistent with this view, loss of adiponectin exacerbates brain aging, leading to heightened anxiety-like behavior and cognitive decline, in part via mitochondrial dysfunction [68]. Moreover, several long-lived murine models, including adipocyte-specific insulin receptor knockout mice, Ames dwarf mice, and growth hormone receptor knockout mice—exhibit persistently elevated adiponectin levels [69–71], reinforcing the notion that adiponectin is positively associated with healthy lifespan.

Epidemiological evidence supports a similar relationship in humans. Centenarians exhibit significantly higher plasma adiponectin levels, which correlate positively with elevated high-density lipoprotein cholesterol (HDL-C) and reduced glycated hemoglobin, and inversely with C-reactive protein and E-selectin concentrations [72]. Elevated adiponectin levels were also observed in centenarians aged ≥95 years, which were negatively associated with body mass index (BMI), waist circumference, and body fat percentage, and positively correlated with HDL-C and lipoprotein particle size [62]. Significantly elevated total adiponectin levels were also observed in 58 Polish centenarians compared with individuals in their 70s [73], further implicating adiponectin as a biomarker and contributor to extreme longevity.

Paradoxically, while preclinical and longevity studies consistently highlight the protective roles of adiponectin, several clinical observational studies report that elevated adiponectin levels are associated with increased mortality in specific pathological contexts, particularly in patients with heart failure, a phenomenon referred to as the “adiponectin paradox.” For instance, Kistorp et al. [74] found that among 195 patients with chronic heart failure, high adiponectin levels were significantly associated with increased mortality risk, independent of BMI or heart failure severity. Similar associations have since been validated in larger cohorts and in patients with ischemic heart disease, type 1 and type 2 diabetes, end-stage renal disease, and even in the general elderly population [75–77]. One plausible explanation for this paradox lies in the concept of “adiponectin resistance.” Mechanistically, adiponectin exerts its effects by binding to its receptors, AdipoR1 and AdipoR2, which activate the AMPK pathway, thereby enhancing cellular energy metabolism [78, 79]. Adiponectin signaling is regulated by APPL1/APPL2 isoforms, which act as integrated Yin–Yang regulators of adiponectin signaling that mediate the crosstalk between adiponectin and insulin signaling pathways in muscle cells [80]. Activation of the AMPK pathway by adiponectin has been found to protect aging-related metabolic diseases, helping to mitigate age-associated metabolic dysregulation [81, 82]. However, in chronic heart failure, Van Berendoncks et al. [83] demonstrated that AdipoR1 expression is downregulated, and PPAR-α/AMPK signaling is impaired, thereby blunting downstream adiponectin signaling despite elevated adiponectin levels. In this context, increased circulating adiponectin may represent a compensatory mechanism in response to impaired energy metabolism, rather than a direct pathogenic driver. In support of this, Mendelian randomization analyses have failed to demonstrate a causal relationship between adiponectin levels and coronary artery disease, suggesting that elevated adiponectin may instead serve as a disease-associated biomarker [84].

Beyond its metabolic actions, adiponectin modulates multiple inflammation-related pathways, including NF-κB, JNK, and MAPK, leading to suppressed pro-inflammatory cytokine expression and alleviation of chronic low-grade inflammation [85, 86]. It also exerts antioxidative effects by reducing reactive oxygen species (ROS) production and enhancing the activity of antioxidant enzymes, ultimately improving mitochondrial function [79, 87]. Taken together, these findings highlight the pivotal role of adiponectin in improving aging and age-related pathologies. Therapeutic strategies aimed at restoring physiological adiponectin levels and preventing adiponectin resistance hold significant potential for promoting healthy aging and mitigating chronic disease burden in the elderly.

### Leptin

Leptin resistance is a common phenomenon in older adults, characterized by elevated serum leptin levels alongside reduced hypothalamic sensitivity to leptin, leading to dysregulation of energy metabolism [61, 63]. As AT ages, it undergoes processes such as inflammation, fibrosis, and depletion of mesenchymal stem cell, which may further exacerbate leptin resistance. Current evidence suggests that leptin resistance may be associated with pro-inflammatory signaling, while chronic inflammation in AT may also contribute to abnormal leptin secretion, creating a vicious cycle [88, 89].

Adiponectin and leptin, as key adipokines, have antagonistic effects. With AT aging, adiponectin secretion decreases, while leptin levels increase, leading to a reduction in the adiponectin-to-leptin (AL) ratio. Studies have shown that a lower AL ratio is positively correlated with BMI, waist circumference, and serum insulin levels, suggesting that impaired adipose function may affect insulin sensitivity and increase the risk of metabolic syndrome [90]. However, while the AL ratio may serve as an important biomarker of AT aging, direct evidence linking leptin to adipose aging remains limited, highlighting the need for further research to better understand the role and significance of leptin in AT aging.

### Plasminogen activator inhibitor-1

Plasminogen activator inhibitor-1 (PAI-1) is a plasma glycoprotein secreted by AT and several other tissues, including endothelial cells, smooth muscle cells, and macrophages [91, 92]. The serum levels of PAI-1 are significantly increased during aging, making it a key marker of AT dysfunction [64]. The role of PAI-1 in adipose aging is primarily linked to its promotion of tissue fibrosis, exacerbation of chronic inflammation, and inhibition of adipocyte progenitor proliferation [93]. As aging progresses, stromal fibroblasts and immune cells within AT secrete elevated levels of PAI-1, which enhances TGF-β signaling, induces collagen deposition, and facilitates ECM remodeling, ultimately reducing AT plasticity [94, 95]. Additionally, PAI-1 activates the p53/p21-mediated senescence pathway, pushing adipocyte progenitors into a senescent state, which impairs adipogenesis and contributes to lipolysis imbalance and the functional decline of AT [96]. Thus, PAI-1 not only serves as a crucial regulator of AT aging but also plays a significant role in mediating aging-related metabolic dysfunction.

### Retinol binding protein 4

Retinol binding protein 4 (RBP4) is a secretory protein produced primarily by AT and the liver, playing a crucial role in the transport of vitamin A (retinol) in circulation. Recent research has expanded our understanding of RBP4 beyond its classical function, revealing its significant involvement in metabolic regulation and aging, particularly in the context of AT aging, inflammation, lipolytic imbalance, and metabolic dysfunction. In obese individuals, RBP4 mRNA and protein levels are markedly elevated in the liver, SAT, and VAT [97]. Moreover, RBP4 expression in SAT is positively correlated with macrophage infiltration and the expression of multiple pro-inflammatory cytokines, suggesting that RBP4 may amplify inflammatory signaling, disrupt AT homeostasis, and impair metabolic function [97]. *In vitro* studies have demonstrated that RBP4 directly promotes basal lipolysis in adipocytes while attenuating insulin’s inhibitory effect on lipolysis. Additionally, RBP4 activates antigen-presenting cells, triggering AT inflammation, and contributing to systemic insulin resistance [98], which exacerbates metabolic dysfunction [97]. During aging, RBP4 levels continue to rise, and its pro-inflammatory and metabolic dysregulation effects may further contribute to AT dysfunction and accelerate AT aging [65]. Given its pivotal role in AT aging, RBP4 has emerged as a potential therapeutic target for metabolic aging-related diseases [65, 99]. Studies suggest that reducing RBP4 levels or blocking its’ signaling pathway can effectively improve AT function, reduce inflammation, and enhance insulin sensitivity [98, 65]. Therefore, targeting RBP4 may provide novel intervention strategies for delaying AT aging and preventing aging-related metabolic disorders [60].

### Fibroblast growth factor 21

Fibroblast growth factor 21 (FGF21), predominantly produced by the liver and to a lesser extent by AT, skeletal muscle, and the pancreas, is a key endocrine regulator of systemic energy homeostasis. It exerts broad metabolic benefits, including reduction of adiposity and hepatic triglyceride accumulation, enhancement of thermogenesis and energy expenditure, and improvement of insulin sensitivity [100, 101]. Studies have revealed that AT-derived FGF21, rather than hepatic FGF21, may regulate beige fat thermogenesis via a paracrine mechanism [102, 103]. Mechanistically, cold-induced upregulation of FGF21 in AT promotes beige fat thermogenesis by stimulating the crosstalk between adipocytes and macrophages via activation of the nicotinic acetylcholine receptor (nAChR) signaling cascade [104]. Importantly, FGF21 exerts anti-inflammatory actions thereby mitigating age-associated chronic inflammation within adipose depots [105, 106]. Circulating FGF21 levels increase with age, but often accompanied by FGF21 resistance, reducing its efficacy in metabolic regulation despite elevated levels [107, 66]. Collectively, these findings position FGF21 as a central node linking metabolic regulation and immune remodeling in AT. Targeting the FGF21 signaling axis holds significant promise for delaying AT aging and ameliorating age-related metabolic dysfunction.

In addition to the aforementioned adipokines, other AT-derived molecules such as resistin, chemerin, and apelin have also been implicated in the aging of AT [108–111]. These secretory molecules not only reflect the physiological state of AT aging but may also serve as potential therapeutic targets for aging-related diseases. Future research should further explore the regulatory mechanisms governing these adipokines to inform the development of targeted interventions aimed at delaying aging and improving healthspan.

## AT dysfunction contributes to organismal aging

AT is among the earliest organs to exhibit functional decline with aging. This dysfunction, characterized by impaired lipid storage capacity in adipocytes and reduced lipolysis in damaged WAT, leads to ectopic lipid accumulation in other tissues, resulting in lipotoxicity [112]. A recent study demonstrated that, compared to young (3-month-old) mice, midlife (12-month-old) mice exhibited significantly enhanced adipogenesis in WAT, accompanied by reduced insulin sensitivity and impaired lipolytic capacity [17]. These changes reflect a shift toward metabolic inflexibility and represent a critical mechanism linking midlife obesity to the onset of metabolic aging [17]. For instance, the accumulation of ectopic lipids in the liver during aging can accelerate the progression of metabolic dysfunction associated fatty liver disease (MAFLD) [113]. A recent study identified WAT as one of the earliest tissues to exhibit aging phenotypes, with some of the most pronounced aging signatures [114].

Aging not only induces widespread activation of immune cells within AT, but this activation is first observed in the white adipose depots of middle-aged mice. Further investigation using single-cell RNA sequencing revealed that immune cells, including T cells and immunoglobulin-producing plasma cells, begin to accumulate in WAT during middle age and subsequently expand to other organs [16]. Comprehensive metabolomic analyses across multiple mouse tissues, coupled with single-cell transcriptomic profiling, have revealed distinct stages of aging. Early aging (3 to 12 months) is characterized by the depletion of metabolism-related cell populations, such as adipocytes and muscle cells, whereas late aging (12 to 23 months) is marked by a significant expansion of immune cell populations. These changes indicate that functional decline in AT begins in the early stages of aging [16].

In addition, a recent study has identified immunoglobulins as key drivers of AT fibrosis and metabolic deterioration, as well as systemic biomarkers of aging [115, 116]. Notably, IgG begins to accumulate in AT early in the aging process, with significantly elevated levels observed in aged WAT. Importantly, interventions targeting IgG accumulation have been shown to mitigate metabolic decline and extend healthspan [115]. This research provides direct evidence that AT is one of the earliest tissues to exhibit aging phenotypes, and targeting its aging processes may significantly delay systemic aging [115].These findings suggest that AT may act as an early initiator of aging.

## AT aging is an initiator of organism aging

**Figure 3.**
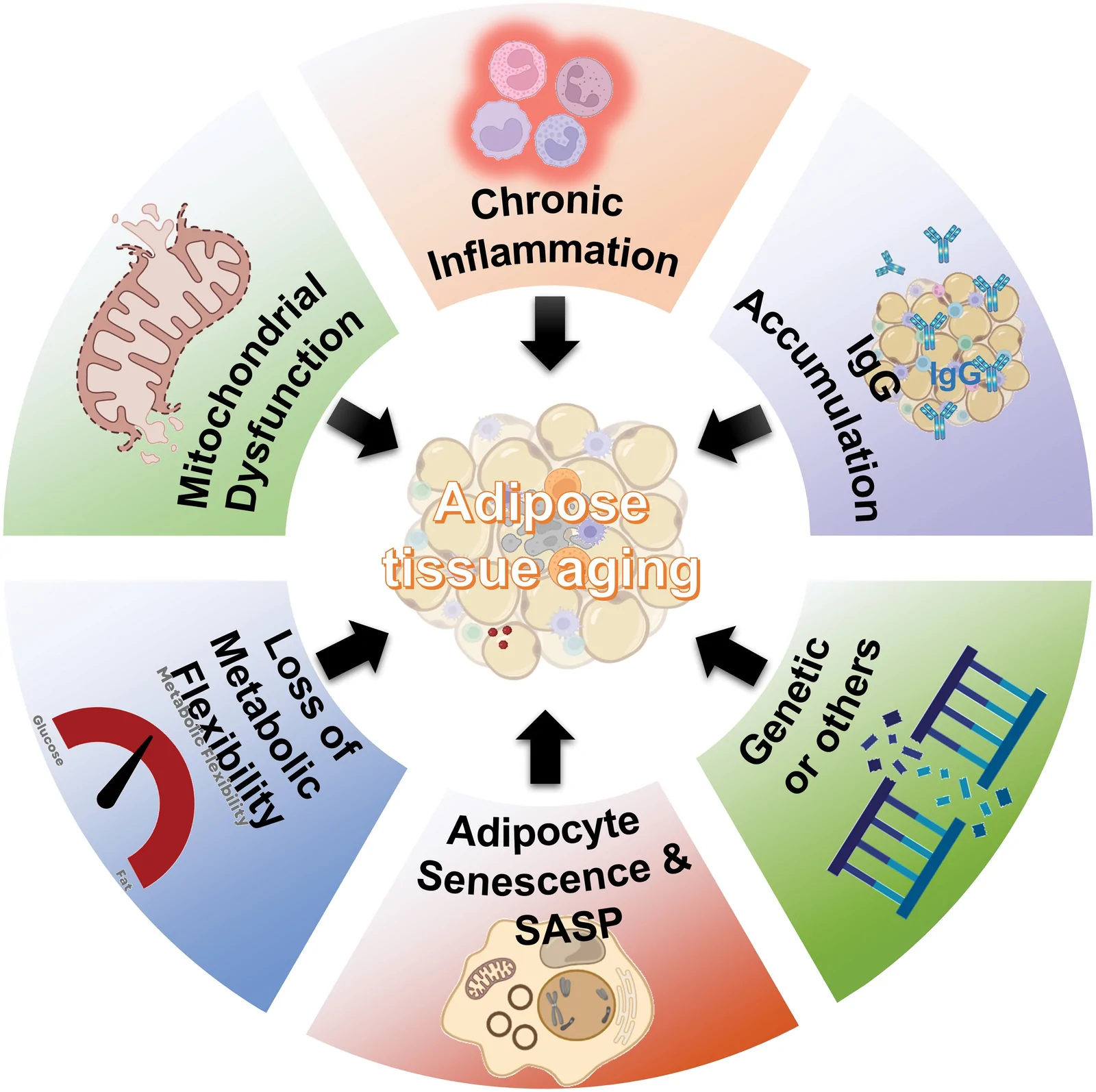
Mechanisms contributing to AT aging. Mitochondrial dysfunction reduces ATP production and increases oxidative stress, promoting adipocyte senescence and metabolic dysregulation. Chronic low-grade inflammation, characterized by the accumulation of pro-inflammatory immune cells and the secretion of inflammatory cytokines, exacerbates tissue dysfunction. IgG accumulation in aging AT further drives fibrosis and immune activation. Reduced metabolic flexibility impairs the ability of adipocytes to switch between lipid and glucose metabolism, contributing to lipid accumulation and systemic metabolic imbalance. Additionally, senescent adipocytes secrete SASP factors, which propagate inflammation and fibrosis. Genetic factors, including alterations in key regulatory pathways such as Nrf2 and SIRT1, further accelerate AT aging by impairing antioxidant defense and mitochondrial function. Created with BioRender.com, released under a Creative Commons Attribution-NonCommercial-NoDerivs 4.0 International license.

AT is one of the most sensitive tissues in the aging process, undergoing extensive changes across biological and physiological pathways that profoundly impact overall health [117]. As aging progresses, AT experiences a progressive decline in adipocyte functionality, structural remodeling, and heightened immune activation [25]. In the early stages of aging, AT’s energy storage and endocrine functions deteriorate [26], resulting in reduced metabolic flexibility [118]. This decline imposes an increased metabolic burden on other tissues, leading to redox imbalance, dysregulated energy metabolism, and accelerated cellular senescence [118]. Age-related redistribution of WAT, particularly the excessive accumulation of visceral, intermuscular, and intramuscular fat, exacerbates systemic metabolic dysfunction due to the distinct metabolic and secretory profiles of adipocytes in these depots. Among these, intramuscular fat is associated with the highest cardiometabolic risk, even surpassing VAT, further contributing to metabolic imbalance. Increased visceral fat also impairs hepatic function by releasing elevated levels of free fatty acids (FFA) and pro-inflammatory factors, which promote insulin resistance and contribute to the development of type 2 diabetes and MAFLD [119–121]. Thus, AT aging not only serves as an early event in aging process but also acts as a key initiator of systemic metabolic disorders and driving inflammaging (Fig. 3).

AT dysregulation contributes to the dysfunction of systemic organs through various mechanisms, thereby accelerating the aging process. Obesity-induced AT hypertrophy is a major contributor to aging. During AT expansion, changes in vascular distribution and oxygen supply are accompanied by ECM remodeling, which is associated with fibrosis [122]. Studies have confirmed that as aging progresses, the degree of fibrosis in AT increases, and ECM remodeling becomes more pronounced [36, 123]. A recent study shows that aging AT exhibits early and significant accumulation of IgG, which induces chronic low-grade inflammation and fibrosis [115]. This fibrosis is a key factor contributing to the metabolic dysfunction associated with hypertrophic growth. Concurrently, fibrosis and ECM remodeling reduce the plasticity and metabolic flexibility of AT, impairing its ability to adapt to metabolic fluctuations and further accelerating systemic aging [37].

AT is a dynamic metabolic organ that constantly adapts to internal and external stimuli, including immune, nutritional, and temperature-related stressors. Chronic energy surplus (e.g. HFDs) and aging-induced reductions in metabolic flexibility impose sustained stress on AT, resulting in lipid accumulation, impaired lipolysis, and diminished adipocyte turnover [124–126]. The reduction in metabolic flexibility further accelerates the progression of AT aging [108]. In addition, AT dysfunction leads to the accumulation of metabolic byproducts, such as FFA, triglycerides, and cholesterol. These metabolic wastes disrupt AT homeostasis and activate immune cells, resulting in chronic low-grade inflammation, a hallmark of aging known as “inflammaging.” This persistent inflammatory state is believed to underlie the systemic pro-inflammatory phenotype associated with aging [127]. AT secretes SASP factors, including inflammatory cytokines, chemokines, and proteases [47, 128, 129]. SASP factors establish a pro-inflammatory environment that exacerbates tissue degradation. Senescent adipocytes and stromal cells residing in AT contribute to tissue dysfunction and systemic inflammation by secreting SASP into neighboring cells [130]. For example, aging AT continuously releases pro-inflammatory cytokines such as IL-6 and TNF-α, inducing chronic low-grade systemic inflammation (inflammaging), which circulates through the bloodstream to affect other key organs. Animal studies have shown that elevated peripheral IL-6 levels are significantly associated with cognitive impairments and the progression of Alzheimer’s disease, likely through mechanisms involving hippocampal microglial activation and exacerbated amyloid-β (Aβ) deposition. In the liver, IL-6 and TNF-α activate JNK and NF-κB signaling pathways, inhibit insulin signaling, and promote lipid accumulation, driving the progression of nonalcoholic fatty liver disease (NAFLD) and its transition to nonalcoholic steatohepatitis (NASH).

Moreover, aging of ADSCs further impairs the tissue’s ability to repair and regenerative. Age-related changes in the characteristics, functionality, and transcriptional profiles of ADSCs ultimately compromise the metabolic capacity of AT [47]. The adipokine profile in aging AT undergoes significant changes, with a reduction in adiponectin levels and an increase in pro-inflammatory adipokines such as leptin and resistin. The reduction in adiponectin not only directly impacts liver lipid metabolism and insulin sensitivity but may also accelerate brain aging. Leptin elevation leads to leptin resistance, further disrupting energy balance and metabolic homeostasis, thereby increasing the risk of neurodegenerative diseases and contributing to the development of fatty liver and insulin resistance through its effects on liver metabolism.

Mitochondrial function is essential for adipocytes to regulate lipid metabolism and maintain energy homeostasis. Recent studies further highlight the pivotal role of mitochondrial dysfunction in AT aging and organism aging. Song et al. [131] demonstrated that Gpx3 deficiency in AT compromises mitochondrial inner membrane integrity, inducing mitochondrial dysfunction. The resultant mitochondrial damage promotes adipocyte hypertrophy and accelerates aging by inducing oxidative imbalance and disrupting systemic metabolism. Moreover, both mitochondrial biogenesis and oxidative phosphorylation are markedly reduced [132]. This decline leads to mitochondrial dysfunction, triggering excessive production of ROS, elevating oxidative stress, and shortening telomeres, ultimately accelerating the senescence of both adipocytes and APCs [133]. These studies demonstrate that mitochondrial dysfunction in AT extends beyond the tissue itself, affecting other organs and playing a decisive role in multiorgan health.

Last but not least, as key endocrine cells, adipocytes secrete a broad spectrum of bioactive factors, including classical adipokines (e.g. adiponectin, leptin) and EVs, that mediate inter-organ communication [134–136]. With aging, AT undergoes significant phenotypic shifts, leading to the release of EVs enriched with microRNAs (miRNAs), cytokines, and protein cargos that modulate the function of distant organs such as the liver and brain. Emerging evidence suggests that AT-derived EVs serve as vehicles for delivering regulatory miRNAs, such as miR-27b, miR-126, and miR-155, to peripheral tissues, thereby regulating gene networks involved in glucose metabolism, insulin signaling, and inflammation [137]. For example, miR-27b delivered via adipocyte-derived EVs impairs hepatic insulin signaling and exacerbates hepatic steatosis by modulating insulin receptor substrate pathways and lipogenic transcription factors [138]. Of particular concern in the context of systemic inflammation and neurodegeneration, miR-155, a pro-inflammatory miRNA enriched in EVs from senescent adipocytes and adipose-resident macrophages, has been shown to promote insulin resistance and inflammatory signaling cascades in hepatic and neural tissues [139]. Beyond miRNAs, AT-derived EVs carry inflammatory cytokines such as TNF-α and IL-1β, which can cross the blood–brain barrier. These cytokine-containing vesicles activate microglia and astrocytes, initiating low-grade neuroinflammation and contributing to age-related cognitive decline and neurodegenerative processes [140]. Thus, aging AT disseminates pathogenic molecular cargos systemically, driving multiorgan dysfunction characterized by metabolic deterioration and neuroinflammation—key hallmarks of organismal aging.

**Figure 4.**
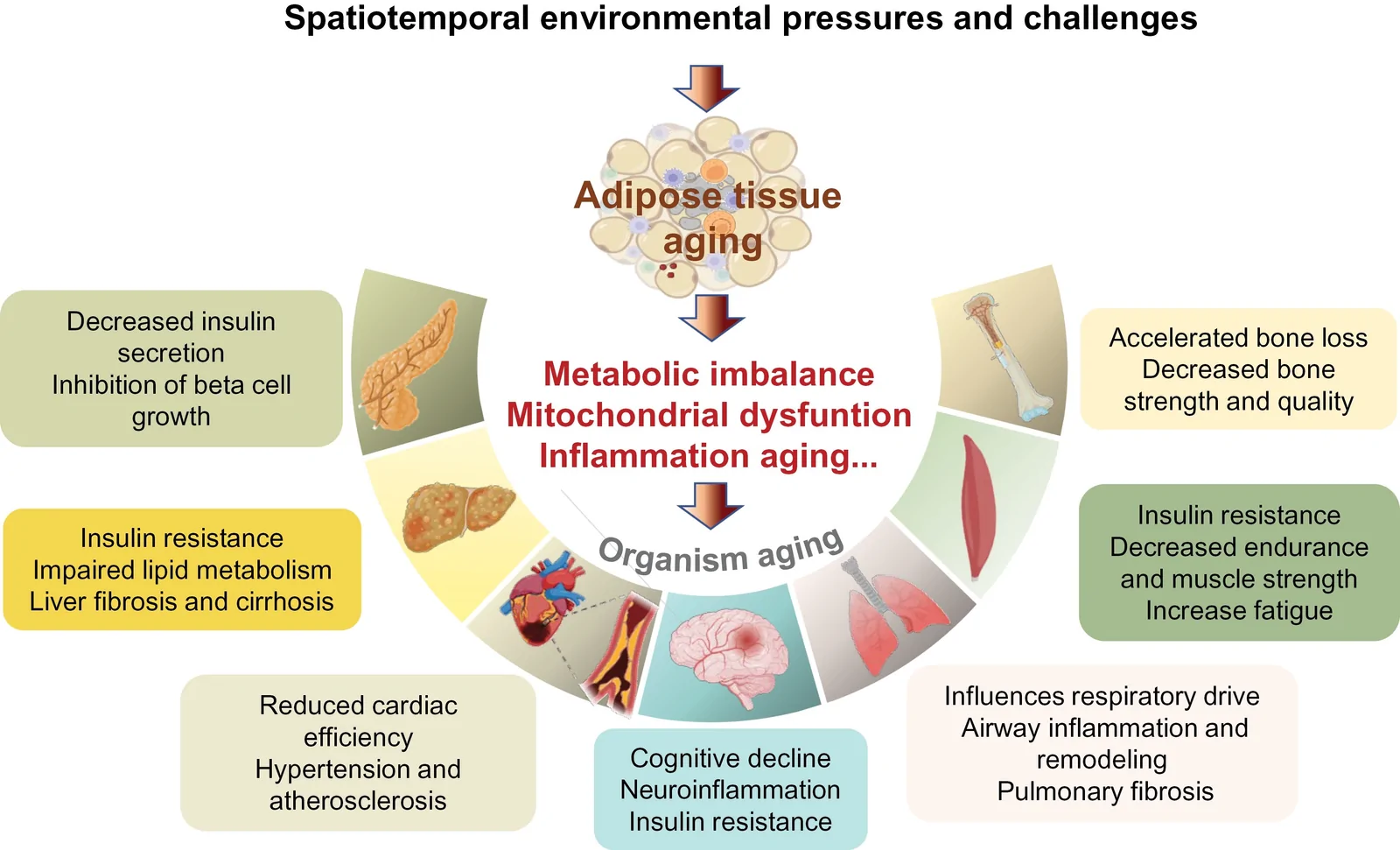
AT aging as a central initiator of systemic aging under environmental and metabolic stress. This figure highlights the pivotal role of AT in driving systemic aging when subjected to environmental pressures and metabolic challenges. The aging of AT leads to systemic dysfunction through key mechanisms, including metabolic imbalance, mitochondrial dysfunction, immune senescence, and chronic inflammation. These pathological processes promote the aging of various organs, including the pancreas (resulting in insulin resistance), liver (fibrosis), cardiovascular system (hypertension, atherosclerosis), brain (cognitive impairment), lungs (chronic obstructive pulmonary disease), skeletal muscle (weakness), and bones (osteoporosis). The progressive degeneration of these organ systems culminates in organismal aging. Created with BioRender.com, released under a Creative Commons Attribution-NonCommercial-NoDerivs 4.0 International license.

In conclusion, aging of AT contributes to systemic aging through multiple mechanisms, including chronic inflammation, dysregulated adipokine secretion, mitochondrial dysfunction, and impaired endocrine signaling. Although the full molecular pathways remain to be elucidated, it is clear that aging AT not only loses its metabolic and hormonal regulatory functions but also actively drives the aging of distant organs via circulating factors (Fig. 4). These findings underscore the central role of AT dysfunction in accelerating organismal aging and highlight its potential as a therapeutic target for age-related diseases [25].

## AT is a key regulator of organism aging

AT plays a dual role as both an early responder and key driver in the systemic aging process. As such, targeting AT aging holds great promise for mitigating age-related diseases and extending lifespan. Numerous studies have highlighted AT as a promising regulator in combating aging. For instance, the expression of genes governed by peroxisome proliferator-activated receptor gamma (PPARγ), a key transcription factor in lipid metabolism, declines progressively with age. In aged mice, low-dose treatment with the PPARγ agonist thiazolidinedione (TZD) significantly reprogrammed gene expression in VAT, notably by suppressing inflammatory pathways. TZD-treated mice showed reduced age-related fat loss, lower levels of inflammation and fibrosis, and improved AT homeostasis [141–143].

Dietary interventions, especially caloric restriction (CR), have also been widely recognized for their ability to delay aging [144, 145]. CR not only prevents age-related abnormal fat accumulation, thereby reducing the risk of damage to nearby and distant organs but also improves AT function through transcriptional reprogramming. This reprogramming involves pathways related to bioenergetics, anti-inflammatory responses, and longevity [31, 146]. Interestingly, CR has been found to maintain PPARγ activity in WAT, thereby preventing the age-related functional decline of AT [144]. These findings suggest that CR exerts antiaging effects by improving AT function, playing a pivotal role in its systemic health benefits.

In addition to dietary interventions, pharmacological approaches have shown substantial antiaging potential. For instance, metformin, a widely used antidiabetic drug, has demonstrated antiaging effects in primates [147]. Mechanistically, metformin appears to exert its effects by upregulating FGF21 expression, reducing abnormal fat accumulation, and improving AT function, thereby mitigating age-related metabolic imbalances [148].

Heterochronic parabiosis, an important model for studying aging, also represents a potential antiaging intervention. Evidence suggests that heterochronic parabiosis can restore immune homeostasis in AT and reverse age-related AT dysfunction [149]. This approach not only improves AT function but also reprograms age-associated gene expression profiles [150, 151]. Additionally, adipose-derived mesenchymal stromal cells, hematopoietic stem cells, and hepatocytes have been identified as among the cell types most responsive to heterochronic parabiosis, potentially playing key roles in rejuvenation [152]. These findings further underscore the critical role of delaying AT aging in antiaging strategies.

Interestingly, genetic interventions targeting adipose-related genes have also been shown to extend lifespan. For example, the expression of the *Nrip1* gene in VAT increases significantly with age, potentially contributing to VAT expansion during aging. In mice, *Nrip1* deletion enhances autophagy, reduces adipocyte senescence and pro-inflammatory cytokine levels, and ultimately extends healthspan [153]. Moreover, deleting Toll-like receptors in mice alleviates age-associated inflammation by reducing processes such as ER stress and cellular senescence, representing a promising antiaging genetic intervention [154]. In addition, targeting AT mitochondrial function and enhancing AT plasticity have emerged as promising strategies for delaying aging [131, 155].

## Potential biomarkers for evaluating aging

### AT volume and distribution

AT redistribution is a hallmark of aging, characterized by subcutaneous fat atrophy, visceral fat accumulation, and ectopic fat deposition in the liver, myocardium, and skeletal muscle. These changes contribute to metabolic dysfunction and increase cardiovascular risk. CT and magnetic resonance imaging (MRI) are the gold standards for quantifying the subcutaneous-to-visceral fat ratio [156]. However, CT exposes patients to radiation, and MRI is costly and less accessible. Dual-energy X-ray absorptiometry (DXA) and ultrasound-based imaging offer potential alternatives for large-scale population studies and longitudinal monitoring.

In aged individuals, subcutaneous fat depletion leads to prominent features such as hollowed cheeks, chin recession, and temple concavities, along with marked reduction in limb fat, particularly in the upper arms and lower extremities. These changes contribute to a frail and cachectic appearance. The decline in the subcutaneous-to-visceral fat ratio is not only a feature of adipose aging but also a biomarker for obesity phenotyping and cardiovascular disease risk stratification [157].

Aging also leads to a progressive decline in BAT volume and activity, with reduced UCP1 expression, impaired mitochondrial respiration, and diminished thermogenic capacity [56]. The decline in sympathetic nervous system activity is a key driver of BAT dysfunction in aging. ^18F^FDG-positron emission tomography (PET)/CT remains the primary imaging modality for BAT activity assessment, but its sensitivity is influenced by ambient temperature, metabolic state, and adrenergic tone, limiting its routine clinical application. Future methodologies may integrate cold-stimulated PET/CT, transcriptomic profiling (e.g. UCP1, PRDM16, PGC-1α expression), and artificial intelligence (AI)-based radiomic analysis to enhance BAT evaluation in aging research.

Despite advances in AT imaging, standardized and clinically feasible methods for adipose aging assessment remain lacking. Future efforts should leverage AI-assisted imaging analytics, multi-omics integration (transcriptomics, proteomics, and metabolomics), and circulating adipose-derived biomarkers (e.g. exosomal miRNAs, adipokines, and lipokines). These approaches will enable noninvasive monitoring of AT aging, improve early risk assessment for metabolic disorders, and enable targeted interventions to mitigate age-associated adipose dysfunction.

### Chronic inflammation in AT aging

Inflammation is a hallmark of AT aging [158, 159], with increased VAT inflammation being an early event in the aging process. A clinical study utilizing single-cell RNA sequencing of SAT from young and elderly individuals identified a distinct PLAU^+^ adipocyte progenitor cell subpopulation, which was significantly expanded in aged individuals and exhibited a pro-inflammatory phenotype [160]. Further supporting this finding, a transcriptomic analysis of human VAT from the GTEx database revealed an age-associated increase in the expression of inflammation-related secretory proteins, underscoring the role of AT inflammation in aging [161].

In animal models, studies have demonstrated that circulating IgG levels are increased with aging in both humans and mice. This results in progressive IgG accumulation in the eWAT of aged mice and in the epicardial fat of elderly individuals. In aged AT, IgG progressively accumulates during the early stages of aging, triggering macrophage-mediated inflammation and inducing TGF-β-mediated adipocyte precursor cell fibrosis. This process contributes to AT fibrosis and metabolic dysfunction. Under obese conditions, IgG exacerbates the metabolic disturbances by directly interacting with the insulin receptor, inhibiting insulin signaling, and promoting insulin resistance [115, 116]. Thus, assessing AT inflammation, particularly through the quantification of infiltrating immune cells and IgG levels, becomes a crucial marker for evaluating AT aging and dysfunction.

SASP refers to the process by which senescent cells actively alter their microenvironment through the secretion of signaling molecules, inducing senescence in neighboring cells and promoting local and systemic inflammation [162]. During AT aging, both adipocytes and immune cells within adipose microenvironment undergo senescence, contributing to a pro-inflammatory SASP profile. Key SASP components, such as IL-1β and TNF-α, play pivotal roles in accelerating systemic aging, impairing AT function, and fostering ectopic lipid accumulation in organs such as the liver and heart, thereby exacerbating the risk of age-related chronic diseases [163].

Beyond the classical SASP factors, emerging evidence points to novel SASP components. For instance, senescent SAT exhibits a significant upregulation of pregnancy-associated plasma protein-A (PAPP-A), which is not only increased in aging AT but also enriched on the surface of EVs secreted by senescent preadipocytes [164]. This highlights the importance of the SASP signature in senescent adipocytes as a crucial biomarker for AT aging, offering valuable insights into its systemic impact and potential avenues for therapeutic intervention.

### AT biopsy

AT biopsy offers a direct approach for assessing the aging status of AT, particularly through SA-β-Gal staining. This method provides valuable high-throughput sequencing data, enabling detailed molecular analysis of AT aging [165]. A prospective cohort study conducted on 227 obese patients demonstrated that biopsies of SAT and omental fat (OF), coupled with colorimetric quantification of SA-β-Gal activity, revealed a significant association between subcutaneous AT aging and abnormalities in glucose metabolism, as well as changes in fat distribution [165]. Therefore, SA-β-Gal staining serves as a robust and quantitative marker of AT aging and is considered the gold standard for evaluating AT aging. In addition, fibrosis in AT is another important indicator of aging, as its progression is closely associated with a decrease in AT plasticity and metabolic function [43, 166, 167]. Therefore, a comprehensive assessment of AT aging can be achieved through SA-β-Gal staining and fibrosis evaluation (Masson staining or Sirius Red staining). This integrated approach offers essential insights for the early screening and intervention in aging-related diseases.

### Lipid profile

Blood lipids encompass the lipid components found in plasma, including cholesterol, triglycerides (TG), FFA, and other lipid molecules. AT aging leads to functional impairment, such as decreased ability to store and mobilize lipids, which in turn results in abnormal lipid levels in the bloodstream. This is typically characterized by elevated total cholesterol (TC), TG, and low-density lipoprotein cholesterol (LDL-C), and reduced high-density lipoprotein cholesterol (HDL-C) levels. These lipid imbalances place additional metabolic stress on tissues and cells involved in lipid metabolism, thereby increasing the risk of aging-related metabolic diseases, such as insulin resistance, fatty liver disease, and cardiovascular diseases [25].

A large survey of 163,641 adults from the China Chronic Disease and Risk Factor Surveillance found that serum TG, TC, and LDL-C levels rise with age, particularly in individuals under 70 years old [168]. Increased TC and LDL-C levels are well-established risk factors for cardiovascular diseases. In a prospective cohort study involving 4.4 million Chinese individuals, higher LDL-C levels were associated with an increased risk of atherosclerotic cardiovascular disease (ASCVD) in both low-risk and primary prevention groups [169]. Furthermore, a decrease in HDL-C levels is also believed to contribute to a higher risk of cardiovascular diseases [170]. Therefore, serum levels of TC, TG, HDL-C, and LDL-C could be considered as potential fluid biomarkers for assessing AT aging, though these markers should be considered alongside comprehensive clinical analysis.

**Figure 5.**
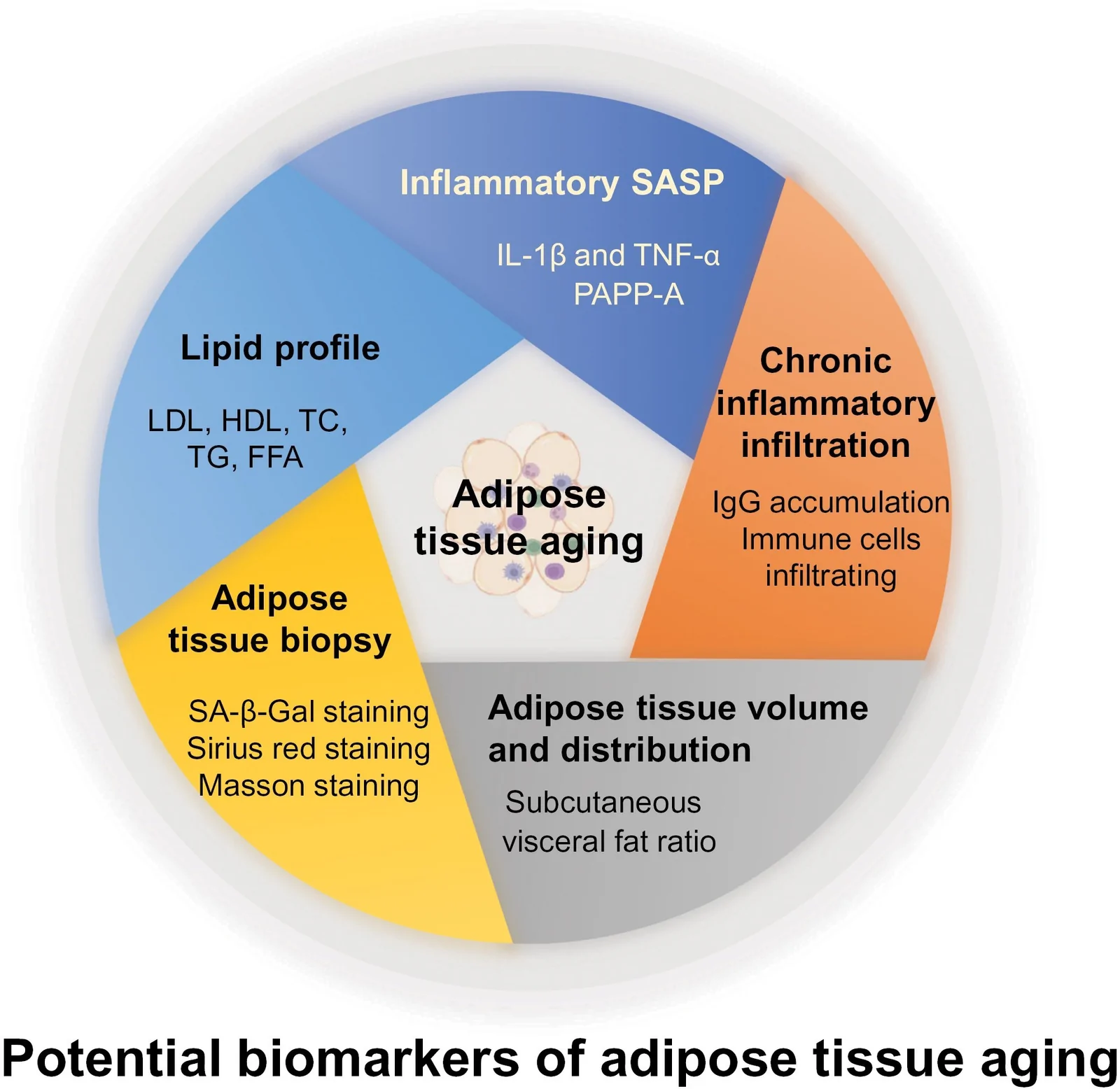
Potential biomarkers for assessing AT aging. This figure highlights key biomarkers that may be utilized to evaluate AT aging. These include inflammatory SASP factors, chronic inflammatory infiltration, AT composition and distribution, AT biopsy findings, and blood lipid profiles. Assessing these biomarkers provides valuable insights into the aging status of AT and its role in aging-related metabolic dysfunction. Created with BioRender.com, released under a Creative Commons Attribution-NonCommercial-NoDerivs 4.0 International license.

In conclusion, these indicators are important biomarkers for evaluating AT aging (Fig. 5). By monitoring these markers, a more thorough assessment of AT aging can be achieved. However, several issues remain. First, there is a lack of a standardized set of biomarkers for AT aging, which limits the precision of such assessments. In the future, multicenter collaborative studies are urgently needed to integrate data from various sources, identify, and validate the most clinically valuable and widely applicable biomarkers. This will ultimately provide more reliable and scientifically grounded methods for the diagnosis and intervention of AT aging.

## Therapeutic strategies for improving at aging

### Senolytics

**Figure 6.**
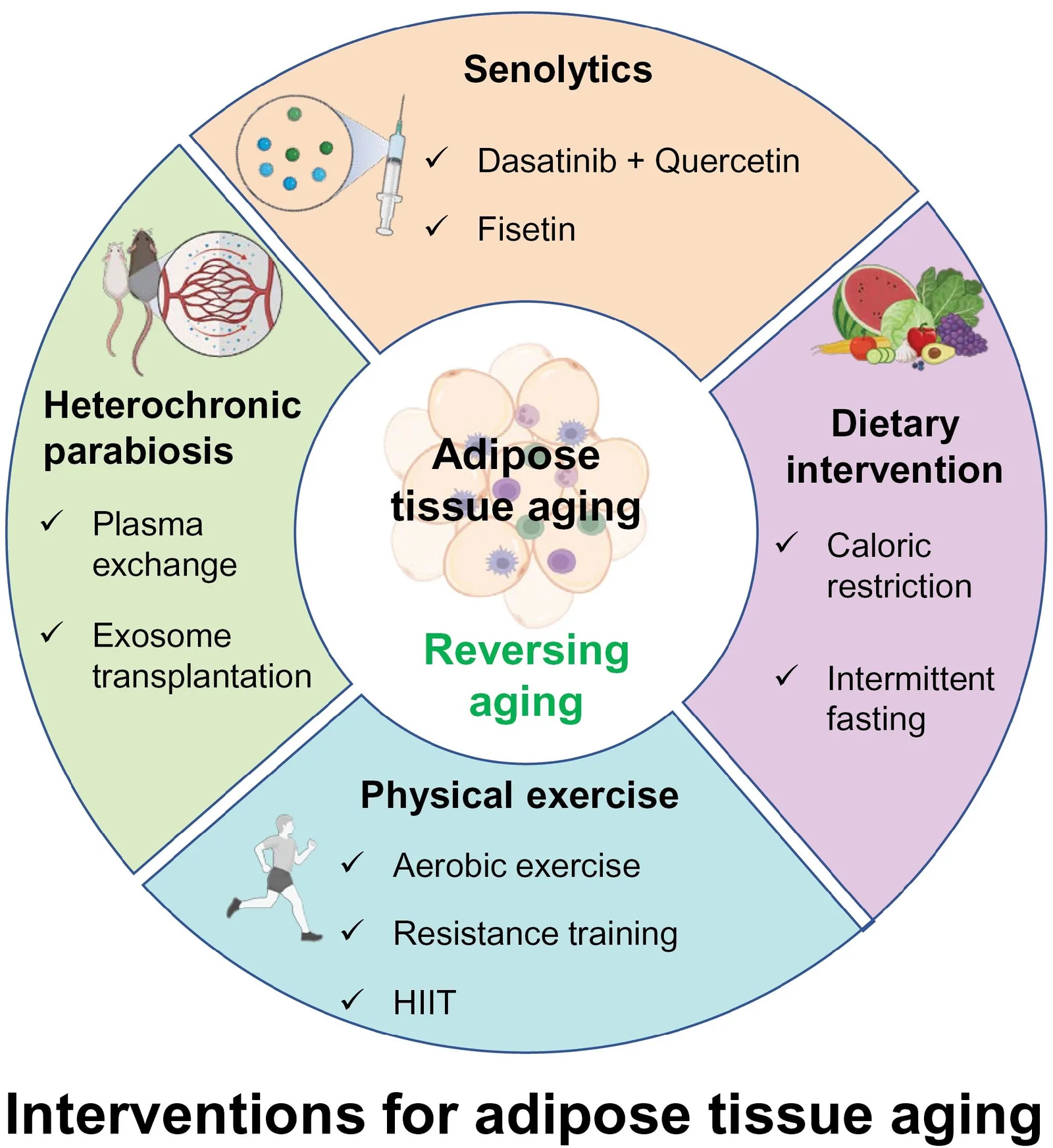
Strategies for targeting AT aging. This figure illustrates four potential therapeutic strategies for combating AT aging. Senolytics selectively eliminate senescent cells within AT, potentially reversing age-related dysfunction. Dietary interventions modulate nutrient intake to regulate metabolic pathways and promote AT health. Physical exercise enhances mitochondrial function and metabolic efficiency in adipocytes, preserving tissue integrity. Heterochronic parabiosis, the transfusion of young blood into aged organisms, has been shown to rejuvenate AT function. These strategies offer promising avenues for mitigating AT aging and its systemic impact on metabolic health. HIIT, high-intensity interval training. Created with BioRender.com, released under a Creative Commons Attribution-NonCommercial-NoDerivs 4.0 International license.

As individuals age, preadipocytes and mature adipocytes in AT gradually enter a senescent state, characterized by a decline in adipocyte function, chronic inflammation (inflammaging), impaired AT remodeling, and disrupted metabolic homeostasis. Senolytics are compounds that selectively target and eliminate senescent adipocytes, thereby removing their pro-inflammatory influence on surrounding tissues, improving AT function, and restoring metabolic balance (Fig. 6). Research on senolytics for AT aging has primarily been conducted on mouse models, with some promising findings emerging from early clinical trials.

Among the senolytic compounds studied, Dasatinib + Quercetin (D + Q) has shown potential in inducing apoptosis in senescent cells by inhibiting the BCL-2 family of proteins. This combination effectively clears senescent adipocyte progenitors, enhances insulin sensitivity, and reduces chronic inflammation [171, 172]. Fisetin, a natural flavonoid antioxidant properties, also inhibits the SASP, targeting AT to promote healthy AT remodeling and mitigate HFD-induced metabolic dysfunction. Navitoclax (ABT-263), which selectively targets BCL-xL to induce senescent cell apoptosis, facilitates the clearance of senescent cells in AT [173, 174]. However, its significant side effects limit clinical application. FOXO4-DRI disrupts the FOXO4-p53 complex and induces apoptosis in senescent cells, thereby promoting healthy AT aging and improving systemic metabolic function (Fig. 6).

Research on senolytics in the context of AT aging has demonstrated great promise, particularly in enhancing insulin sensitivity, reducing AT inflammation, and restoring metabolic homeostasis [175]. Several clinical trials have begun translating senolytic therapy into human studies. In particular, a pilot Phase I trial evaluating the intermittent oral administration of D + Q in individuals with idiopathic pulmonary fibrosis demonstrated good tolerability and potential systemic benefits, including a reduction in senescent cell burden. Another ongoing trial (NCT04785300) is assessing the effects of D + Q in patients with early stage Alzheimer’s disease. Although these studies do not directly target AT, they offer critical proof-of-concept that senolytic therapy is feasible in humans and may provide multitissue benefits. Moving forward, clinical trials specifically designed to target adipose senescence are essential to determine tissue-specific efficacy and evaluate the impact on metabolic health.

### Dietary intervention

Dietary interventions have been extensively studied as nonpharmacological approaches to delaying AT aging, regulating metabolic function, and reducing the accumulation of senescent cells. Current research on dietary strategies targeting AT aging primarily focuses on CR and intermittent fasting (IF) (Fig. 6).

CR is the most widely studied dietary intervention for aging and has demonstrated longevity benefits across multiple species, including humans [176]. A 2-year randomized controlled trial has shown that CR significantly reduces subcutaneous, visceral, and intermuscular fat in older adults. Building on these findings, the ongoing CALERIE 2.0 study (NCT05651620) is evaluating the long-term effects of CR on biological age, metabolic health, and aging-related biomarkers. Additionally, long-term CR (20%–40% CR) has been shown to reduce the expression of senescent gene in AT [177], lowers pro-inflammatory cytokine levels, such as IL-6 and TNF-α, and alleviate systemic inflammation [178]. Additionally, CR helps preserve the function of ADSCs, delaying the decline in AT regeneration and improving metabolic regulation [179]. CR also enhances autophagy and mitochondrial function, reduces oxidative damage in aging AT, and thus improves energy metabolism [31, 180, 181]. However, despite its antiaging potential, long-term adherence to CR remains challenging, which limits its clinical applicability.

In contrast, IF has gained increasing attention as a more flexible dietary strategy. A 10-week IF intervention in 18–20-month-old mice, which corresponds to approximately 60 years of age in humans, leads to reduced fat accumulation, and induces cellular remodeling in visceral fat [182]. IF reduced senescent phenotypes of adipose stem and progenitor cells, restoring their adipogenic potential [182]. It also modulates mTOR and AMPK signaling, promoting autophagy in adipocytes and facilitating the clearance of damaged organelles, such as mitochondria, thereby reducing AT inflammation [183, 184]. IF has also been shown to enhance adiponectin levels [185], improve insulin sensitivity, and mitigate age-related AT dysfunction [186–188]. Additionally, time-restricted feeding (TRF) has been found to activate BAT and increase UCP1 expression, promoting thermogenesis and energy expenditure [189]. Clinical studies suggest that IF may improve metabolic health and slow AT aging, particularly in individuals with metabolic disorders, such as type 2 diabetes [190]. However, its long-term safety and effects on aging individuals require further investigation.

Overall, dietary interventions hold significant promise in regulating AT aging. Strategies such as CR and IF have shown varying degrees of efficacy in mitigating AT senescence and metabolic decline. However, the adaptability of these approaches, their long-term safety, and the mechanistic underpinnings of these dietary patterns require further investigation. Several clinical studies are currently investigating the effects of various dietary interventions on healthspan and aging biomarkers. For example, trial NCT05549362 is examining the influence of traditional and adaptive CR, as well as traditional and adaptive TRF, on key mechanisms of aging. Insights from these ongoing trials are expected to shed light on how dietary strategies influence AT function, metabolic health, and the overall aging process. Ultimately, these findings will provide a stronger scientific foundation for the development of evidence-based, antiaging dietary interventions.

### Physical exercise

As a well-established nonpharmacological intervention, exercise exerts profound effects on AT aging by promoting AT remodeling, suppressing inflammation, improving mitochondrial function, and modulating adipokine secretion [191–195].

Studies have demonstrated that exercise promotes the browning of WAT by upregulating UCP1 and PGC-1α, increasing mitochondrial density, and shifting adipocytes toward enhanced fatty acid oxidation and thermogenesis [196]. Additionally, exercise activates BAT, enhancing its thermogenic capacity and energy expenditure [197], which supports energy balance and reducing the risk of aging-related metabolic diseases. Regular aerobic exercise, such as running and swimming, has been found to stimulate modulate key metabolic signaling pathways, including Wnt/β-catenin, AMPK, and PGC-1α, thereby improving AT metabolic plasticity [198–200]. These adaptations enhance the balance between energy storage and utilization while reducing lipotoxicity-associated metabolic stress. In addition, exercise effectively attenuates SASP-driven chronic inflammation [201]. Regular physical activity promotes the phenotypic switch of pro-inflammatory M1 macrophages to anti-inflammatory M2 macrophages, reducing levels of TNF-α and IL-1β while increasing the secretion of IL-10, a key anti-inflammatory cytokine [202]. Exercise also stimulates β-adrenergic receptor-mediated lipolysis, thereby reducing the burden of senescent adipocytes and alleviating AT inflammation, ultimately restoring immune homeostasis [203]. Besides, as a potent modulator of mitochondrial function, exercise activate PGC-1α, SIRT1, NRF1, and TFAM [204, 205], promoting mitochondrial biogenesis and enhancing adipocyte energy metabolism.

It is also important to note that exercise plays a pivotal role in regulating the endocrine function of AT [194]. It has been demonstrated that exercise upregulates beneficial adipokines such as adiponectin, FGF21, and irisin, which are involved in maintaining glucose and lipid homeostasis, promoting fatty acid oxidation, and enhancing insulin sensitivity [194, 206].

In summary, exercise contributes to delaying systemic aging and maintaining metabolic health. Future research should focus on optimizing exercise modalities, developing individualized exercise regimens, and explore the potential synergies between exercise and senolytics to develop targeted strategies for addressing AT aging, ultimately enhancing healthspan and longevity.

### Heterochronic parabiosis

Heterochronic parabiosis is a classical model in aging research that involves the surgical fusion of the circulatory systems of young and aged organisms to investigate the effects of bloodborne factors on tissue homeostasis and aging. The model has revealed widespread beneficial effects of young blood on aged tissues, including the muscles, bones, heart, and brain of aged mice [151, 207–210]. Recently, Brigger et al. [149] demonstrated that heterochronic parabiosis can reverse age-related dysfunction of WAT by restoring immune homeostasis within AT. Additionally, heterochronic transplantation has been found to reverse aging-associated gene expression signatures [151, 208].

The Tabula Muris Senis project recently identified adipose mesenchymal stromal cells, hematopoietic stem cells, and hepatocytes as cell types particularly sensitive to heterochronic parabiosis [211]. Studies further suggest that soluble blood factors, rather than blood cells, play a predominant role in restoring youthful function [212, 213].

Sahu et al. [214] demonstrated that the beneficial effects of young blood on aged skeletal muscle regeneration are mediated by circulating EVs. Furthermore, Ghosh et al. [215] using a murine heterochronic parabiosis model, an *in vitro* stromal vascular fraction culture system, and a 3T3 preadipocyte culture system to provide evidence that young blood circulation reverses cellular senescence and the pro-inflammatory phenotype of aged AT.

In summary, interventions targeting AT, ranging from lifestyle modifications (e.g. CR), to pharmacological treatments (e.g. TZD and metformin) and gene-editing technologies, can significantly improve health, delay aging, and mitigate age-related diseases (Fig. 6). Future research should focus on elucidating the mechanisms underlying AT aging and identifying key targets for intervention to develop more effective antiaging strategies.

## Future directions

AT is now recognized not merely as an energy reservoir but as a dynamic organ involved in metabolic regulation, immune modulation, and systemic aging. Although significant progress has been made in understanding the role of AT in aging, important knowledge gaps remain—particularly regarding the depot-specific characteristics of aging AT, such as the functional role of beige fat in human aging. One of the most promising areas in AT aging research lies in the development of early diagnostic tools and biomarkers. Early detection of AT aging could enable timely, personalized interventions to prevent or delay the onset of age-related conditions, including obesity, cardiovascular disease, and type 2 diabetes.

Growing new evidence suggests that aging AT serves as a key initiator of systemic metabolic aging. However, the interconnections between AT aging and the aging of other organs, such as the liver, brain, and muscle, remain largely unexplored. Specifically, the mechanisms through which aging AT communicates with distant organs via secreted adipokines, EVs, and other signaling factors require further investigation. Unraveling these pathways will be essential for deepening our understanding of systemic aging and may open new avenues for targeted interventions to delay or mitigate age-related decline.

Finally, while aging is inevitable, the central challenge in aging research is how to promote healthy aging. In the context of AT, although its function declines with age, certain beneficial capacities are retained. This raises a critical question: how can we preserve the healthy functions of AT while preventing pathological hypertrophy, inflammation, and functional deterioration? Addressing this question is essential for reducing the risk of age-related diseases and improving overall metabolic health during aging.

In conclusion, future research on AT aging will aim to clarify its unique characteristics, develop early detection tools, identify targeted interventions, and explore how aging AT interacts with other organs. Through interdisciplinary and cross-sector collaboration, scientists are well positioned to uncover the complex mechanisms driving AT aging and to develop new strategies for preventing and treating age-related diseases.

## Conclusion

In conclusion, AT, as a pivotal endocrine organ, plays a central role in the aging process. It is well-established that AT is an early responder to aging that significantly contributes to the progression of systemic aging. Targeting AT aging holds promise as a strategy for delaying the onset of age-related processes and mitigating their impact.

However, despite significant progress, many aspects of the relationship between AT and aging remain poorly understood. In particular, the mechanisms through which AT acts as an initiator of aging and drives systemic aging processes have not yet been fully elucidated. Given that AT is among the first organs to exhibit age-related changes, it is crucial to develop objective and comprehensive methods for assessing its aging status. Additionally, there is an urgent need for more longitudinal studies to better understand the temporal dynamics of AT dysfunction during aging, which will aid in the identification of novel biomarkers for the early detection of AT aging. As such, future research into AT aging is of paramount importance.
